# Kindergarteners Use Cross-Situational Statistics to Infer the Meaning of Grammatical Elements

**DOI:** 10.1007/s10936-022-09898-0

**Published:** 2022-07-06

**Authors:** Sybren Spit, Sible Andringa, Judith Rispens, Enoch O. Aboh

**Affiliations:** Amsterdam Center for Language and Communication, University of Amsterdam, Spuistraat 134, 1012 VB Amsterdam, The Netherlands

**Keywords:** Statistical learning, Miniature language, Language acquisition, Functional elements

## Abstract

Many studies demonstrate that detecting statistical regularities in linguistic input plays a key role in language acquisition. Yet, it is unclear to what extent statistical learning is involved in more naturalistic settings, when young children have to acquire meaningful grammatical elements. In the present study, we address these points, by investigating whether statistical learning is involved in acquiring a morpho-syntactic structure from input that resembles natural languages more closely. We exposed 50 kindergarteners (*M* = 5 years, 5 months) to a miniature language in which they had to learn a grammatical marker that expressed number, and which could only be acquired on the basis of the distributional properties in the input. Half of the children performed an attention check during the experiment. Results show that young children are able to learn this meaning. We found no clear evidence that facilitating attention to the input increases learning performance.

## Introduction

Acquiring a language encompasses several seemingly distinct tasks: learners need to detect words within a continuous stream of speech, they need to map meanings onto these words and they have to group these words into abstract categories and determine the grammatical relations between these categories. There are several theories on how learners achieve these steps: scholars working from a modular point of view have argued that processing words involves a different cognitive capacity than acquiring grammatical rules (e.g., Berwick & Chomsky, 2015; Chomsky, 1965; Pinker, 1994, 1997), whereas others have suggested that domain-general processing capacities, that are possibly not specific to language, enable learners to grasp the linguistic regularities in all stages of acquisition (e.g., Bybee & McClelland, 2005; Tomasello, 2003; Ullman, 2016). Some argue that such a domain-general ability to learn language relies on the capacity to detect distributional properties within language (e.g., Romberg & Saffran, 2010; Erickson & Thiessen, 2015; Frost & Monaghan, 2016).

The process of extracting information from distributional properties in the input is often labeled statistical learning. Statistical learning has been demonstrated to play an important role in visual learning (e.g. Baker, et al., 2004; Bertels et al., 2015), but it has also been suggested to contribute to language acquisition. Yet, it has been argued that statistical learning capacities cannot solely explain how languages are acquired (Lidz & Gagliardi, 2015; Yang & Montrul, 2017). The question is rather to what extent statistical learning is involved in the acquisition process. In this paper, we addressed this question by exposing children to more naturalistic input than is typically used in statistical learning experiments. Additionally, as a more exploratory question, we investigated whether raising attention to the input of the language children have to learn, influenced their learning outcomes.

## Statistical Learning Experiments

Studies within the artificial grammar learning paradigm have shown that tracking statistical regularities allows learners to recognize word boundaries and establish dependencies between linguistic elements (e.g. Endress & Bonatti, 2007; Gomez & Gerken, 1999; Perruchet & Vinter, 1998; Saffran et al., 1996; Thiessen et al., 2013). In such studies, participants are typically confronted with a carefully designed stream of artificial linguistic input, with certain elements co-occurring and others not. After exposure, participants are tested on their ability to distinguish between stimuli that co-occurred in the input and stimuli that did not co-occur systematically. If participants were able to track the statistical dependencies between co-occurring elements during exposure, they should be able to recognize those stimuli during such a test. Results suggest infants detect such regularities (Aslin et al., 1998), and it has also been shown that they are able to generalize acquired regularities to novel items, when co-occurring elements are not directly adjacent (Gomez, 2002). It is important to note that, unlike in natural languages where auditory input possesses meaning, the statistical regularities in these studies did not carry meaning.

Recent evidence seems to indicate that statistical learning also plays a role in form-reference mappings (e.g. Frank, et al., 2009; Frank et al., 2013; Kachergis et al., 2014; Smith et al., 2014; Vouloumanos, 2008; Yu & Smith, 2007). This has been established using cross-situational statistical word referent learning tasks, in which participants have to acquire nonsense words and their meanings. Instead of tracking which linguistic sounds co-occur together, learners now have to grasp the statistical dependency between certain sounds and particular visual stimuli. In a typical trial, participants are exposed to multiple pictures (e.g. a dog and a cat) simultaneously and hear the accompanying nonsense words (e.g., *bovo* and *pano*) in a sound stream. In a subsequent trial, they are presented with a novel combination of pictures (e.g. a horse and a cat) and matching words (e.g., *pano* and *orbo*). Although it is impossible to learn which word refers to which picture from a single trial, this can be accomplished across multiple trials, if a participant is able to detect that a certain nonsense word (*pano*) frequently occurs in combination with a particular picture (a cat). Studies report that infants are able to learn word meanings in such tasks (e.g. Smith & Yu, 2008) and that, as they grow older, they are capable of learning word meanings when the interval between word referent pairs increases (Vlach & Johnson, 2013).

Furthermore, studies like those by Lany (2014) and Lany and Saffran (2013) indicate that distributional properties in the input help learners to determine to which semantic category words belong. They showed this using cross-situational learning tasks in which referents were divided into two categories (e.g., animals and vehicles) and words that referred to them were accompanied by different suffixes/particles (e.g., *erd* and *alt*) or had different syllabic structure (e.g., mono- and bi-syllabic). At test, participants were exposed to a new word that had the same characteristics as words they encountered during training (*erd* and mono-syllabic) and were asked to match this new word to a picture of a particular category (animals). Participants succeeded to link the right properties to the right category (i.e., that mono-syllabic words refer to animals). In these experiments, participants would thus show learning when they were able to detect the statistical association between linguistic elements and corresponding abstract semantic categories. Similar experiments have also shown that learners can use such information to categorize nouns and verbs (Monaghan et al., 2015), to group objects according to their shape (Chen et al., 2017), and to determine which words in the input are function words and which are not (Hochmann et al., 2010).

Studies in the lab have further shown that learners can acquire more complex grammatical patterns aided by the distributional properties of the input (e.g. Goldberg et al., 2004; Samara et al., 2017; Wonnacott et al., 2017). In these studies, participants were exposed to English sentences containing a novel grammatical structure that they could map to a novel meaning. For example, a sentence with a novel verb and two English nouns in a subject-object-verb order would mean that the subject appeared on or into the object in the manner denoted by the verb (Goldberg et al., 2007). The statistical regularity participants had to grasp here was the association between a particular word order and a specific verbal meaning. At test, participants heard the grammatical construction with another novel verb, and saw two pictures of which one contained the intended meaning. Participants showed learning by mapping this word order to the correct picture. Similar studies show that five- to seven-year old children learn the grammatical form better when a single verb occurs more frequently in the new construction than others (Casenheiser & Goldberg, 2005), and that in six-year-olds the knowledge of phrasal constructions is stronger when they are retested a couple of days later (Wonnacott et al., 2012). Importantly, studies of this kind are typically not classified as ‘statistical learning studies’. Although the properties of the input are manipulated in these experiments, they involved incorporating a novel structure into the native language of participants, instead of exposing participants to a language in which all lexical items are completely new. The exact statistics of the input are therefore not controlled as meticulously as in statistical learning studies, but nevertheless still present learners with input that does not resemble naturalistic input.

## Functional Elements in Artificial Languages

The statistical learning studies discussed above often vary considerably in terms of the statistical information that learners need to draw on: from tracking co-occurrence between sounds (e.g. Endress & Bonatti, 2007; Gomez & Gerken, 1999; Saffran et al., 1996) to matching a word order pattern to a verbal meaning (e.g. Goldberg et al, 2004; Wonnacott et al., 2017). However, they all share some important commonalities. In order to acquire the investigated linguistic structures, learners needed to associate particular stimuli with each other. Whether these stimuli were syllables, word orders, or abstract categories, participants acquired the linguistic structures by tracking the statistical dependency between these stimuli in artificial settings. It does not seem unreasonable to assume that one domain-general capacity for statistical learning is involved in these different learning tasks (Romberg & Saffran, 2010). Statistical learning has thus been demonstrated to be involved in tracking word boundaries, learning words and their referents, but also in grouping words in larger semantic categories and in acquiring more complex grammatical structures.

Yet, languages do not consist of only lexical categories but also involve functional categories (e.g., demonstratives, adpositions, tense, mood, aspect markers) which not only convey specific meaning but determine how the lexical category they combine with must be interpreted. The following examples (1) and (2) illustrate the contribution of such grammatical elements, in English and Gungbe. In English, a noun that refers to a countable entity cannot occur in a sentence by itself, but needs to be combined with a determiner. This can be a definite article, as in (1a), which enforces the interpretation of ‘table’ as known to both speaker and hearer, or a demonstrative as in (1b), which denotes proximity to the speaker.

- ‘Koku bought the table’

1. ‘Koku bought *this* table’

In Gungbe, a Gbe language of the Kwa family, which is spoken in the Bight of Benin, we encounter a different situation. This language allows bare nouns to occur in a sentence, with an interpretation that could be (in)definite singular, generic, or generic plural (i.e., a/the table, (some) tables, or tables in general). Only the context allows speakers to tease those different interpretations apart (cf. Aboh, 2004; Aboh & DeGraff, 2014). In this language, however, a bare noun (2a) must be distinguished from a noun accompanied by the grammatical element *lɛ́* (2b), which forces the interpretation of the noun as plural and definite.

**Table:** 

(1a)	*Kɔ̀kú*	*xɔ̀*	*távò*
	Koku	buy-perf	table
	‘Koku bought a/the table’		
	‘Koku bought (some) tables’		

**Table:** 

(1b)	*Kɔ̀kú*	*xɔ̀*	*távò*	*lɛ́*
	Koku	buy-perf	table	numb.def
	‘Koku bought the tables’			

When comparing these English and Gungbe examples therefore, we see that the grammatical elements not only determine the grammatical pattern but also contribute to the meaning of that pattern. Indeed, proximity, definiteness, and number are not inherent semantic properties of the nouns *távò* and *table*. These meanings are added through the grammatical elements the nouns co-occur with, and learners need to grasp this association to interpret these sentences correctly. An important point here is that while lexical items can refer to entities or events that have some representation in the actual world and in such cases allow acquisition of their semantics, the semantics of grammatical items can only be acquired through context. We therefore wanted to know whether statistical learning is involved in acquiring grammatical or functional elements using a miniature language learning experiment.

## Current Study

This study is not the first to investigate whether young children acquire this type of grammatical structure in an artificial language learning setting. Culbertson and Newport (2015) for example investigated whether children would prefer a particular word order when learning a grammatical marker. They exposed children to pictures of objects, and combinations of modifying marker and a noun describing the picture. These combinations could occur in two different orders (e.g. noun-marker and marker-noun), one of which would be more frequent in the input. Input frequency did not matter for learning the meaning of the modifier, but was sometimes in line with what would be predicted from linguistic universals, and sometimes not. The researchers found that when children saw a picture and had to describe this, they overgeneralized the more frequent word order for their descriptions only when this was in line with what linguistic universals would predict. Using a comparable kind of experimental set-up, Tal and Arnon (2020) show that children are able to learn an optional plural marker, but that children are also less likely to use this optional marking for noun classes that occurred as plurals in the input infrequently than for noun classes that occurred as plurals frequently. Furthermore, Raviv and Arnon (2018) found in an iterated learning experiment, that children could learn a similar grammatical element expressing plurality from exposure to noun phrases, even when linguistic input was relatively unstructured, because earlier generations of learners produced unstructured output.

Although these studies show that young children are able to learn meaningful grammatical elements from limited linguistic input in various artificial language learning settings, Culbertson and Schuler (2019) pointed out that the languages used in many of these studies are rather different from what natural languages look like. Studies like those described above, for example, present children with merely noun phrases (e.g. Culbertson & Newport, 2015; Raviv & Arnon, 2018; Tal & Arnon, 2020) or computer synthesized speech streams (e.g., Gomez & Gerken, 1999; Saffran et al., 1996). Other studies incorporated new grammatical structures in the language that children already spoke (e.g. Goldberg et al., 2004; Samara et al., 2017; Wonnacott, et al., 2017) or exposed children to completely novel visual stimuli (Smith & Yu, 2008; Vlach & Johnson, 2013).

Crucially, to see whether learners acquire grammatical elements that bear a meaning relying on statistical learning in more natural settings, it is important to test them using an experimental procedure where the input resembles a natural language more closely than in typical artificial language learning experiments. Some studies addressed this issue already. For example, Yurovsky, et al., (2013) let adults participate in a cross-situational word learning study where they saw real life shots from parent child interaction. In an infant study, Lew-Williams, et al., (2011) varied utterance length, such that it resembled child direct speech more closely. We aim to extend these experiments by investigating whether statistical learning is involved in more naturalistic settings, when young children have to acquire a meaningful grammatical element.

In the present experiment, kindergarteners were therefore trained on an artificial language in which a grammatical marker combines with nouns to express number, to investigate whether they use statistical information from the input to acquire a meaningful grammatical element. In our language nouns could be preceded by two markers: *pli* and *tra*. The marker *tra* was a default nominal marker with no number specification, whereas *pli* indicated that the noun always refers to multiple referents. Statistical properties of the input were the only cue for acquiring this meaningful grammatical element, which resembles the marker that is discussed in (2).

To learn this regularity, children were exposed to a miniature language containing novel lexical items of which the statistics were carefully manipulated, and that resembled a natural language more closely than studies we described earlier. The marker itself is one that is similar to markers that occur in natural languages, as mentioned before. Furthermore, the language not only contained noun phrases to acquire the grammatical marker, but also verbs, proper names and conjunctions, which were not necessary for acquiring the grammatical marker. Children could learn the meaning of the marker pictures of objects and animals familiar to children at this age, which were drawn in a children book like style. At test, children were presented with a picture matching task in which number was the crucial feature to distinguish between possible answers. Because the grammatical marker *tra* was not indicative of number, whereas *pli* was, we expected learning the grammatical marker would lead to better performance for test items including *pli* than for test items including *tra.* Thus, our experiment was designed in such a way that we could answer the question whether distributional properties of the input allow children to acquire a grammatical element that expresses number in a language that resembles a natural language more closely than previous artificial grammar learning experiments. Half of our participants performed a check that raised attention to the linguistic input, to investigate whether the inclusion of an attention check would lead to higher learning rates. Such a finding might be relevant for future studies that use this experimental set-up.

## Method

### Participants

50 native, mono-lingual, Dutch speaking children (25 males, 25 females, *M* = 5; 5 years, *SD* = 0;10, *range* = [4;2–7;1]) took part in this experiment. All children were in kindergarten and were recruited from a primary school in the city center of Haarlem. The school is located in a neighborhood that had transitioned from an average socio-economic status to a relatively high socio-economic status in the years prior to data collection (SCP, 2016). The experiment was conducted during a set number of days at the school, and as many children as possible within these days participated in the experiment. All children were able to participate in the full experiment. Their teachers reported that none of the children were diagnosed with any language or communication disorders. No restrictions were imposed upon taking part in the experiment. No further information about the children’s (non-)linguistic background was collected.

### Materials

#### Miniature Language and Target Structure

To place the children in a naturalistic learning environment, an artificial language was created that consisted of four proper names, three verbs, two grammatical markers, one conjunction, six frequent nouns, and twelve infrequent nouns, which were necessary for designing test items (see below). All words and their translations can be found in Table 1. Apart from the proper names, which might occur in Dutch, all words were novel words, which were loosely based on word forms from Esperanto. The chosen forms were sometimes slightly manipulated such that all word forms within a particular word class were equally likely to be Dutch words, based on the transitional probabilities between phonemes within each word. As a result, words within a particular class had similar structural properties, which prevented some words being learned more easily than others.

**Table 1:** All words from the miniature language and their translations

Word type	Word	Translation
Proper name	*Carlo*	‘Carlo’
	*Julia*	‘Julia'
	*Marco*	‘Marco’
	*Maria*	‘Maria’
Verbs	*Estima*	‘Looking’
	*Pentura*	‘Taking a picture’
	*Rigarda*	‘Eating’
Frequent nouns	*Domo*	‘Tree’
	*Herbo*	‘Banana’
	*Kego*	‘Horse’
	*Lito*	‘Flower’
	*Pano*	‘Cat’
	*Zambo*	‘Apple’
Infrequent nouns	*Ando*	‘Carrot’
	*Anso*	‘Dress’
	*Arbo*	‘Castle’
	*Bovo*	‘Strawberry’
	*Halto*	‘Rabbit’
	*Kobro*	‘Sheep’
	*Misto*	‘Dog’
	*Nego*	‘Egg’
	*Nutro*	‘Painting’
	*Teko*	‘Sandwich’
	*Wiro*	‘Car’
	*Wolgo*	‘Cow’
Grammatical marker	*Pli*	‘Plural’
	*Tra*	‘Any number’
Conjunction	*Ut*	‘And’

The language had subject-verb-object word order, like Dutch has in main clauses. In the miniature language, a noun phrase on its own does not encode number and could correspond to both singular and plural referents. In a sentence, however, an argument noun phrase must be introduced by the nominal marker *tra*. The type of nominal marker included in our artificial language is quite common in languages with residual noun classes, such as the Kwa-language Akan (Appah, 2003) or the Austronesian language Cebuano (Parnes, 2011). In our miniature language, a noun introduced by *tra* can refer to both singular and plural referents: the correct interpretation must be grasped from the visual context. The sole function of *tra* therefore is to turn a bare noun into an argument. The language includes another nominal grammatical marker *pli*, which encodes number and indicates that the noun necessarily refers to multiple referents. Apart from carrying information about definiteness, the function of this marker is similar to the previously discussed marker from Gungbe. The marker *tra* can be seen as a default nominal marker with no number specification, whereas *pli* not only turns the noun into an argument but adds information about number. In short, the rule participants had to learn was that whenever *pli* preceded a noun, this noun always referred to multiple referents. Dutch does not contain such a number marking grammatical element. Instead Dutch nouns are pluralized through suffixation of the noun (Booij & van Santen, 2017), which is comparable to English pluralization.

#### Structure of the Experiment

**Fig. 1:**
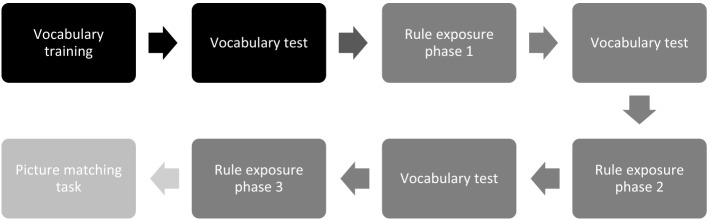
A visual representation of the structure of the experiment. The darkest boxes show the parts of the vocabulary training. The lighter boxes show the different parts of the rule training. The lightest box shows the test phase

Before describing the precise characteristics of the input to learn this grammatical regularity in the next section, we will briefly lay out the overall structure of the experiment. An overview of this structure can be seen in Fig. 1. To learn the regularity, participants were told a story with four protagonists (Carlo, Julia, Marco and Maria), who were going on a holiday to a country of which they did not speak the language. Participants were asked to help the protagonists learn this new language. They were told they would see pictures and hear things in the new language that matched the pictures they saw. The experiment consisted of three parts. It started it out with a short vocabulary training. After the vocabulary training a rule training session followed. During this session, children received input to acquire the grammatical rule. After the rule training, participants performed a test phase to test whether they acquired the rule.

As we were interested in the acquisition of the grammatical rule and function, and not in that of lexical vocabulary items, we bootstrapped participants slightly into learning the lexical items by means of a short vocabulary training on the six frequent nouns of the language only. In this training, the nouns were presented auditorily, without the grammatical markers *pli* or *tra* accompanying them, and with a picture showing the meaning of the corresponding bare noun. Each noun was presented six times during this training, twice with one, twice with two and twice with three referents, to make sure a bare noun would not be associated with a particular number of referents. After this training, participants were given a picture matching task to test their vocabulary knowledge. They heard one of the frequent nouns and saw four pictures with referents of the other frequent nouns. Participants could progress to the next phase when they correctly identified each frequent noun four times. After three incorrect responses for a certain noun, the experimenter would provide feedback for the correct answer. When a particular noun had not been identified correctly four times, it would continuously reoccur in the vocabulary test, until four correct answers were reached for that noun as well, in order to make sure all participants would have the same starting point when beginning to learn the rule. The length of the vocabulary training could thus vary across participants, but this would not have consequences for the amount of input to the target rule, as the vocabulary training did not expose participants to the functional elements.

After the vocabulary training, participants were exposed to three rule training phases in which they received input on the basis of which they could learn the grammatical rule. During each phase, 40 sentences were presented, adding up to a total of 120 sentences. Each sentence was accompanied by a picture showing the meaning of the sentence. A subset of 108 sentences consisted of a proper name, a verb, a marker and a noun. In these training sentences, the proper name functioned as subject, while the noun was the object. These sentences served as the input to learn the plural marking rule. The other 12 sentences were created using two proper names, the conjunction and a verb. These sentences did not contain a noun and thus did not provide evidence of the grammatical rule that had to be learned. These sentences were used to create the attention check (see below) and must be regarded as fillers. Examples of all sentence types and their accompanying pictures can be found in Table 2. Sentences in the language were always semantically plausible. After rule training phases one and two, participants were given a six item vocabulary test (one item for each frequent noun), using the same procedure as described earlier. These vocabulary tests were inserted to maintain participants’ attention. Participants would receive a sticker after each vocabulary test, regardless of their results.

**Table 2:** Examples of artificial language training sentences and rough translations. For sentences with *tra* the noun could be translated both as a singular and a plural. The correct interpretation should be grasped from the visual context

	Example	Translation	Picture
*Tra* + single referent	*Maria rigarda tra zambo*	‘Maria eats (an) apple(s)’	
*Tra* + multiple referents	*Carlo estima tra pano*	‘Carlo looks at (a) cat(s)’	
*Pli* + multiple referents	*Julia pentura pli anso*	‘Julia takes a picture of dresses’	
Filler	*Marco ut Maria pentura*	‘Marco and Maria take a picture of each other’	

Furthermore, because previous studies indicate attention plays a critical role in statistical learning (Arciuli, 2017; Toro et al., 2005), we wanted to know whether the inclusion of a task to maintain attention would lead to higher learning rates. 24 of the 50 participants performed this attention check. In the attention check condition, the four protagonists sometimes could not hear what had been said in the new language correctly. This was indicated by a questioning face of a protagonist after a certain stimulus. When participants saw this face, they had to repeat the previously heard stimulus. This attention check was inserted to keep participants focused; they did not have to repeat what had been said correctly. Participants were introduced to this attention check during the vocabulary training where they had to repeat each noun once. During the rule training phase, they had to repeat four filler sentences per training phase. Filler sentences did not contain a grammatical marker and a noun, and occurred at fixed moments during each phase. Participants who did not perform the attention check also heard these filler sentences, but simply did not have to repeat them. There was no difference in input for the rule, nor salience of this input, between the two conditions.

#### Characteristics of the Input

From the 108 training sentences that participants could use to learn the rule during the rule training phases, 72 sentences contained the default marker *tra*, while 36 sentences contained the marker *pli*, which was indicative of number. In half of these 72 *tra* sentences, the noun had one referent. In the other half, the noun had multiple referents. In half of the sentences that contained a noun with multiple referents, two referents were shown, in the other half of the sentences that contained multiple referents, three referents were shown. This distribution was the same for sentences with *pli* that showed multiple referents and *tra* that showed multiple referents. As a result, 36 pictures showed one referent, 36 pictures showed two referents, and 36 pictures showed three referents. Furthermore, the probability that the noun following *pli* had multiple referents was 1. For a noun following *tra*, this probability was 0.5, as the probability that it referred to a single referent was 0.5 as well. Vice versa, when a single referent was shown, the probability of hearing *tra* before the noun was also 1. If a participant saw multiple referents, the probability of hearing *tra* before the noun was 0.5, as was the probability of hearing *pli* before the noun.

Every frequent noun occurred twelve times in the input. It occurred four times with *pli* and eight times with *tra.* When a frequent noun occurred with *tra*, it referred to a single referent four times and multiple referents the other four times. Every infrequent noun occurred three times in the input, once with *pli* and twice with *tra*, of which it once referred to a single referent and once to multiple referents. Each noun referred to one, two or three referents equally often. Furthermore, when nouns were plural, they occurred equally often with *pli* as with *tra.* Every noun occurred equally often over each of the three rule training phases, as did every grammatical marker. An overview of the characteristics of the input can be found in Table 3.

**Table 3:** Different types of sentence in the input and how often they occurred per block and with each noun type

Structure	Total occurrences	Occurence per noun type	Picture
*Tra* + single referent	36 times 12 times per block	4 times per frequent noun 1 time per infrequent noun	
*Tra* + multiple referents	36 times 12 times per block	4 times per frequent noun 1 time per infrequent noun	
*Pli* + multiple referents	36 times 12 times per block	4 times per frequent noun 1 time per infrequent noun	
Filler	12 times 4 times per block	Did not contain a noun	

#### Test Phase

Immediately after the rule training phase, participants took part in a picture matching task to determine whether they became sensitive to the grammatical cue. In this task, participants heard 36 sentences based on the twelve infrequent nouns from the rule training phase, and had to choose which of two pictures matched each sentence. Infrequent rather than novel nouns were used, so participants would feel a target response could be based on what they had been exposed to. Young children reportedly have difficulties with tests in which they have to make decisions that are unrealistic to them (for a methodological review, see Pinto & Zuckerman, 2018), which would be the case when test items contained novel nouns and pictures. We tried to circumvent this by using infrequent rather than novel nouns in the picture matching task. This way, participants could base their response on what they had been exposed to; both pictures would be realistic options, as they would have seen them. Yet, by using infrequent nouns, we could still maximize the chance these children used rule knowledge when giving an answer, as chances are slim that they learned the meaning of these nouns from only three occurrences in the input.

24 out of 36 sentences used during the test phase were experimental items. For experimental items, participants had to choose between pictures with either one or multiple referents. The two pictures always referred to different referents. Twelve experimental items contained *pli*. For these items, the target picture always showed multiple referents. Another twelve experimental items contained *tra*. Any number of referents would be grammatical for these items. For half of the experimental items containing *tra*, the target picture showed multiple referents. For the other half of these items, the target picture showed a single referent. Every noun occurred once with *tra* and once with *pli* during the test. If participants learned the meaning of the infrequent noun, they should produce a target answer in both conditions. The performance on the sentences with *tra* can be used as a baseline to which we can compare performance on the sentences with *pli*. We hypothesized that sensitivity to the statistical regularity in the input would lead to more target answers on trials with *pli* than on trials with *tra*, because for trials with *pli* participants could base their response on the number of referents the picture showed, whereas the number of referents was not indicative for trials with *tra*. For all test items, distractors were other infrequent nouns.

In addition to the 24 test items, 12 filler items were included. Fillers contained *pli* or *tra*, but showed the same number of referents on both pictures. These fillers were included to avoid that participants would link the grammatical markers to number during the test phase only, because they always had to choose between a picture with multiple and a picture with a single possible referent. Examples of the different types of items can be seen in Table 4. Items of the different types were presented in a counterbalanced semi-randomized order: we randomized the list of test items, and then moved items containing the same infrequent noun to a different place so that these would not occur consecutively.

**Table 4:**
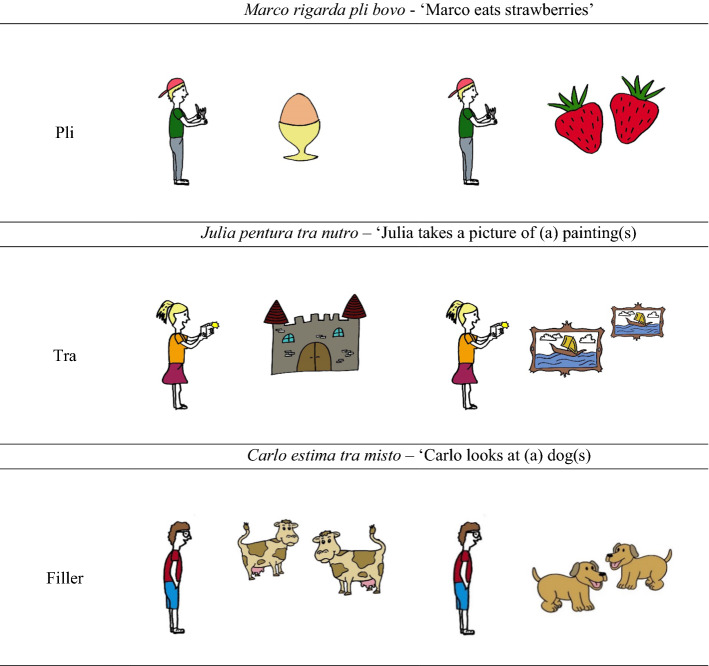
Examples of test items and their rough translations. For sentences with *tra* the noun could be translated both as a singular and a plural. The correct interpretation should be grasped from the visual context. In all three examples, the right picture was the target

After the picture matching task, a debriefing took place in which we examined whether participants were aware of their knowledge in a way they could verbalize. During this debriefing, participants were asked how they knew what the correct answer during the picture matching task was, and whether they knew the meaning of the words *pli* and *tra*.

### Procedure

All stimuli were recorded by a female native speaker of Dutch. The test was administered in a quiet room at the participants’ school. The task was presented on a laptop using E-prime (Psychology Software Tools, Pittsburgh, PA). During vocabulary training, nouns and their accompanying pictures would be presented for three seconds, before automatically moving to the next noun. During rule training, sentences and their accompanying pictures would be presented for four seconds, before automatically moving to the next sentence. During the test phase, the experimenter pressed a button on the keyboard that corresponded to the answer the participant gave (z for the left, m for the right picture). Scores from this phase were registered automatically. Both the participant and the experimenter listened to the audio using headphones. The vocabulary training lasted 8 min on average, the rule training phase 15 min, and the test phase 5 min. The full experiment took approximately 30 min per participant. Ethical approval for this study was obtained from the Ethics Committee of the Faculty of Humanities of the University of Amsterdam (file number 2017–14) and opt-out consent was obtained from children’s parents or legal guardians before the start of the study (i.e. children were allowed to participate, unless their caregivers objected to participation). Full materials can be found on our OSF page (Spit, et al., 2019a).

## Results

To determine whether participants grasped the target regularity and whether the attention check had an effect on task performance, a generalized linear regression model with mixed effects and orthogonal sum-to-zero coding was carried out. Using this analysis, we investigated whether children’s statistical learning ability enabled them to learn a grammatical marker based on distributional patterns in the input. If they were indeed able to do so, we expected them to give more target answers on trials with *pli* than on trials with *tra* during the test phase, because for trials with *pli* participants could base their response on knowledge of this marker, whereas such grammatical knowledge was not indicative of a target answer for trials including *tra*.

**Fig. 2:**
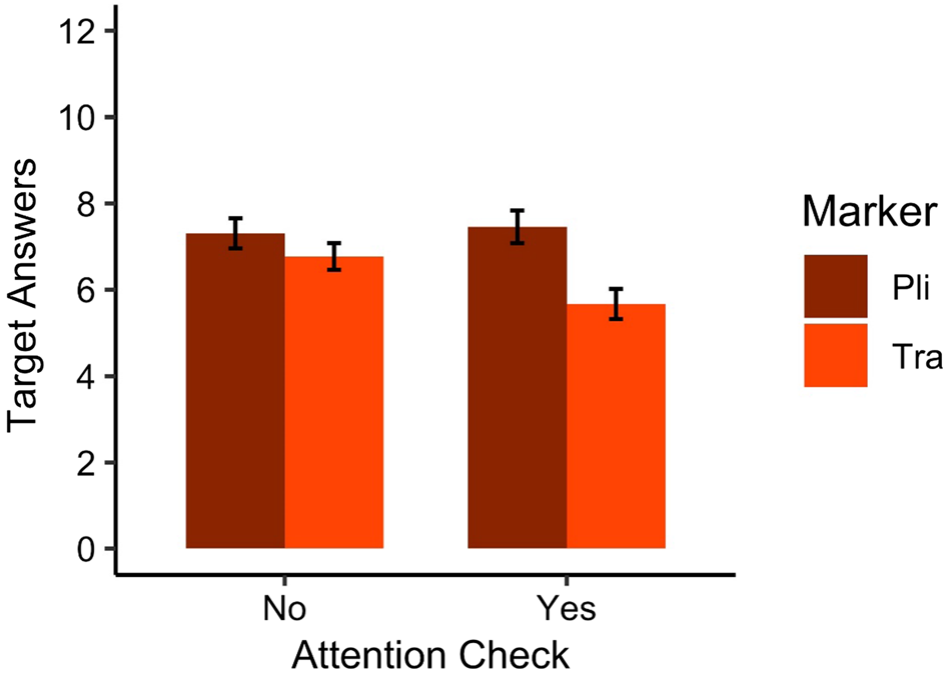
Graph depicting the results from the picture matching from children who did not perform the attention check (left) and children who performed this task (right). The latter group of children were asked to repeat some of the filler sentences during training. Scores indicated the number of target answers produced and could range from 0 to 12. Error bars represent the SE of the mean number of target answers produced. Providing more target answers for sentences containing *pli* than for sentences with *tra* could be seen as indicative of learning the grammatical marker

This analysis was carried out in R (R Core Team, 2015) using the lme4 package (Bates, et al., 2015) where needed. All analyses are published on our OSF page. The generalized linear regression model took the responses from the picture matching task (1 for target, 0 for non-target) as a dependent variable, marker type as a within-participants fixed effect, attention check as between-participant fixed effect, participant as a between-participants random effect and item as a within-participants random effect. Marker type was included as a random slope for participant, because it was a within-participant fixed effect. Attention check was included as a random slope for item, because it was a between-participant fixed effect. Our fixed effects were included in this model, because we were a priori interested in their contribution to the outcome (Gelman & Hill, 2007).^1^ Orthogonal sum-to-zero contrast coding was applied to our binary fixed effects (i.e., marker type and attention check; Baguley, 2012, pp. 590–621). As we aimed to keep the model as fully specified as possible (Barr et al., 2013), we increased the number of possible iterations to 100 000 (Powell, 2009), to solve issues of non-convergence. This enabled us to report on a maximal random effect structure justified by our data (Jaeger, 2010). We report simple rather than standardized effect sizes (Baguley, 2009) and Wald confidence intervals (Agresti & Coull, 1998). The descriptive statistics for this analysis can be found in Table 5 and Fig. 2. Results from the generalized linear regression model showed that participants gave more target answers when sentences contained *pli* than when sentences contained *tra* (*OR* = 1.515, 95% CI = [1.042, 2.201], *z* = 2.178, *p* = .029), suggesting they were sensitive to the statistical regularity in the input.^2^ Results did not show a significant main effect of attention check (*OR* = 0.847, 95% CI = [0.645, 1.112], *z* = -1.197, *p* = .231). The interaction between the attention check and learning the marker was not significant (*OR* = 1.552, 95% CI = [0.965, 2.5496], *z* = 1.824, *p* = .070).

**Table 5:** Scores from the picture matching task indicating the number of target answers produced. Sentences with grammatical marker *pli* were predictable. Scores could range from 0 to 12

	With attention check (*n* = 24)			Without attention check (*n* = 26)			Combined (*n* = 50)		
	M	SD	Range	M	SD	Range	M	SD	Range
*Pli*	7.46	1.86	4–11	7.31	1.76	5–12	7.38	1.79	4–12
*Tra*	5.67	1.71	3–10	6.77	1.58	4–10	6.24	1.72	3–10

Possibly, this response pattern was due to a plurality bias in the children. They could have given more correct answers for *pli* because of a preference for objects with multiple referents. To check for such a bias, we ran an additional analysis, in which we divided the test items containing *tra* in two groups based on whether the target picture showed one or more objects. We conducted a generalized linear regression model, which took the responses from the picture matching task (1 for target, 0 for non-target) as a dependent variable, and target type (singular or plural) as a within-participants fixed effect, participant as a between-participants random effect and item as a within-participants random effect. Target type was included as a random slope for participant, because it was a within-participant fixed effect. This analysis showed no evidence that children gave more correct answers when the target picture showed multiple objects than when it showed one single object (*OR* = 1.002, 95% CI = [0.559, 1.797], *z* = 0.008, *p* = .994). Thus, there is no reason to assume children had a plural bias when providing answers in the picture matching task. Note that it was also unlikely that children gave more correct answers for sentences with *pli*, because of a possible familiarity effect; sentences with *tra* were twice as frequent in the experimental input than sentences with *pli*.

**Fig. 3:**
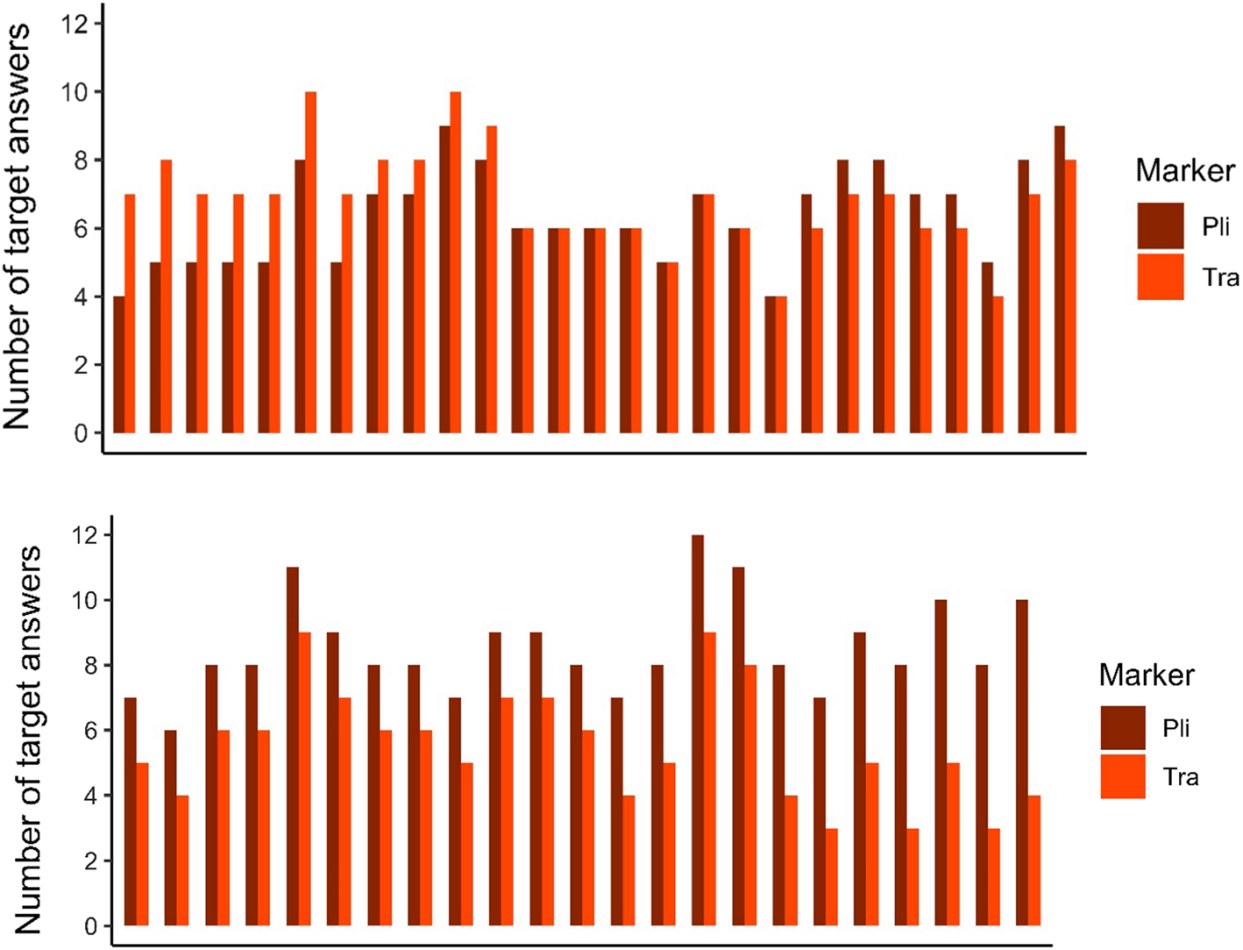
Graphs depicting the number of target answers produced during the picture matching task per participant, ordered by the size of their difference score. The number of target answers for both markers could range from 0 to 12. In total, 62% of the participants (*N* = 31) exhibit a positive difference score, 16% of the participants (*N* = 8) a neutral difference score, and 22% of the participants (*N* = 11) a negative difference score. The graph is split in half for ease of inspection

To shed further light on children’s individual differences in learning the grammatical marker, we calculated a learning score for each child. As input for this score we used the number of target answers for sentence with *pli* and sentences with *tra* of every individual participant, which can be observed in Fig. 3. We calculated the learning score by subtracting the number of target answers on sentences with *tra* from the number of target answers on sentences with *pli*. A larger difference in a positive direction indicates that the participant gave more target answers on sentences with *pli* than on sentences with *tra*. Difference scores per participant for the picture matching task can also be observed from Fig. 3. In total, 62% of the participants (*N* = 31) exhibit a positive difference score, 16% of the participants (*N* = 8) a neutral difference score, and 22% of the participants (*N* = 11) a negative difference score.

During the exit interviews, none of the participants was able to report on the regularity. When asked how they came to a decision on the picture matching task, participants either reported they did not know how they made a decision (74%, *N* = 37), or they claimed that they had heard the sentences before and remembered what they meant (26%, *N* = 13). When asked for the meaning of *pli* and *tra*, children did not know the meaning of these words (80%, *N* = 40), provided the meaning of noun that they were exposed to (18%, *N* = 9) or came up with a completely new meaning (2%, *N* = 1).

## Discussion

The results from this cross-situational statistical learning experiment show that kindergarteners were able to detect a regularity of a number feature encoded by a grammatical marker. As the distributional properties in the input were the only cue to detect the regularity, this study indicates that young children have a discovery procedure that makes them sensitive to such grammatical regularities. We hypothesize that these learners might employ statistical learning mechanisms to tackle the problem of acquiring a grammatical marker that also carries meaning. Previous studies have already shown that statistical learning plays a role in word segmentation (e.g., Aslin et al., 1998; Endress & Bonatti, 2007; Gómez, 2002; Gómez & Gerken, 1999; Perruchet & Vinter, 1998; Saffran et al., 1996; Thiessen et al., 2013), word referent learning (e.g., Frank et al., 2009, 2013; Kachergis et al., 2014; Smith & Yu, 2008; Smith, et al., 2014; Vlach & Johnson, 2013; Vouloumanos, 2008; Yu & Smith, 2007) and the acquisition of semantic categories (e.g., Chen et al., 2017; Lany, 2014; Lany & Saffran, 2013; Monaghan et al., 2015). Although the statistical information that learners needed to use varied considerably between these studies, learners always had to associate particular pieces of information with each other. What had been studied very little, is whether this is the same with grammatical markers that express meaning, such as markers of nominal number. The present study therefore tested whether kindergarteners could grasp a statistical dependency between an auditory linguistic element and a visual plural interpretation of this marker. Importantly, we investigated this in a miniature language learning experiment, using input that resembled natural linguistic input more closely than earlier studies that investigated similar grammatical structures (e.g. Raviv & Arnon, 2018; Tal & Arnon, 2020).

Indeed, the results are compatible with the idea that a domain-general capacity for statistical learning plays a role in learning such meaningful grammatical elements. However, we should note that, although this learning effect (i.e., the difference between *pli* and *tra* sentences) was significant, it was relatively small. One explanation for this finding might be that children received only a relatively limited amount of input, which is not comparable to what they would receive in a natural learning environment. Indeed, a more robust learning effect might be observed if children would have more time to learn the language, but practically this is very difficult to achieve within such an experimental setting: the length of the current experiment was already at the limits of what is feasible with children from this age group. Alternatively, the small learning effect might mean that statistical learning is involved in detecting the distributive properties of a grammatical element, but interacts with other learning mechanisms that lead to acquisition of the meaning (e.g. Lidz & Gagliardi, 2015; Yang & Montrul, 2017). A final explanation for the small learning effect might be that children used noun knowledge during test rather than rule knowledge. However, we do think this is unlikely since every infrequent noun in the test phase occurred once with *tra* and once with *pli*. If participants learned the meaning of a particular infrequent noun, they should have produced a target answer in both conditions, and we should not have observed a difference score.

The results also might indicate that learning this type of regularity slightly improved when participants performed a task intended to increase their attention, since we observed a bigger difference in target answers between *pli*- and *tra*-sentences for children who participated in the attention check, than for children who did not participate in the task. However, evidence for this is far from conclusive as the effect did not reach significance, and because it mainly seems to be driven by the fact that children who participated in the attention check gave less target answers for sentences containing *tra*. Yet, if this finding was indicative of better learning, this would be in line with earlier studies that indicated that statistical learning might benefit from extra attention (e.g. Arciuli, 2017; Toro et al., 2005). The inconclusive outcomes with regard to the role of attention in our study could be a result of the small main effect of learning we found, or some sort of measurement error. Alternatively, it might indicate that attention plays some role in statistical learning, but that its role is relatively small. It could also be the case that our current task did not fully capture attention of the participants, because it was not sufficiently distinct from the learning task at hand. Replications of this study are highly necessary to elucidate to what extent statistical learning and attention are exactly contributing factors when learning meaningful grammatical elements.

Furthermore, responses from the exit interviews also seem to be in line with the idea that statistical learning happens without any awareness involved (e.g. Arciuli & Simpson, 2012; Aslin et al., 1998; Baker et al., 2004; Kidd, 2011; Reber, 1967; Rebuschat, 2013). Not a single child could report on the plural marking rule that was present in the input. However, these results should be interpreted with some caution, as awareness might exist at different levels. Learners may possess phenomenal awareness (when they can verbalize their experiences) or could be aware at the level of access (when they cannot verbalize or remember their experiences coherently; Cleeremans, 2008, 2011, 2014). A verbal report as used in this experiment taps only into the former type of awareness (Timmermans & Cleermans, 2015) and is thus inconclusive about the involvement of awareness of the latter type. Perhaps, participants in this experiment were aware of the regularity in a way they could not verbalize. To establish whether such awareness is involved in statistical learning, a novel kind of experimental method would be required, a method that would capture awareness even if children are not able to verbalize their awareness (see Spit et al., 2019a; 2019b, for an example of such a method).

Several other factors may have influenced how well the marker was learned, as we observed considerable individual differences in learning scores. For some children, the difference between *pli-* and *tra-*sentences was quite large, even though they did not score very well overall. Some children scored highly on *pli*-items, which suggests learning of the marker, but also scored well on *tra*-items. All kinds of factors not measured in this study could cause such behavioral differences. For example, children may vary in how easily they pick up the meanings of words, there may have been effects of knowing or speaking other languages besides Dutch, or children could vary in general cognitive capacities. The goal of the current study was to investigate whether children could learn this type of marker, and not necessarily whether certain factors make some children better learners than other children. Therefore, we chose not to collect any data about the (non-)linguistic background of these children, to keep the sample representative of the population of interest (cf. Kruskal & Mosteller, 1979; Kukull & Ganguli, 2012 for discussions about different types of representation). This choice, however, does not enable us to explore if and how particular individual characteristics might be related to the observed individual variation in our sample. Future research could thus delve deeper into the driving forces behind these individual differences.

In sum, this study provides evidence that statistical learning might support the acquisition of a meaningful grammatical element. However, it is important to note that participants in this experiment had to learn a grammatical marker with a relatively simple abstract meaning. Natural languages also exhibit more complex abstract grammatical patterns than the rule that was presented in our artificial language. Whether statistical learning mechanisms can account for the acquisition of more complex patterns remains unknown, let alone more complex patterns in the wild. Further research is needed to gain a better understanding of the role of statistical learning in language acquisition, especially because it is difficult to determine to what extent statistical learning studies in the lab scale up to naturalistic language learning environments. If more complex patterns can be acquired through statistical learning as well, this would give further reason to assume this learning mechanism could be able to support a broad array of language acquisition processes. This study is a small, but important step into that direction.

## Footnotes

1 The code for this model was thus as follows: Score ~ MarkerType * AttentionTask + (MarkerType | Subject) + (AttentionTask | Item).

2 We also ran two one-sided t-tests to compare scores on both sentence conditions to chance (50%). Results show that participants perform better than chance on sentences containing *pli* (*t*(49) = 5.44, *p* < .001), whereas performance on sentences with *tra* does not significantly differ from chance (*t*(49) = 0.99, *p* = .165).

## References

[CR1] Aboh EO (2004). The Morphosyntax of Complement-Head Sequences. Clause Structures and Word Order Patterns in Kwa.

[CR2] Aboh EO, DeGraff M, Åfarlí TA, Maehlum B (2014). Some notes on bare noun phrases in Haitian Creole and Gungbe. The Sociolinguistics of Grammar.

[CR3] Agresti A, Coull BA (1998). Approximate is better than “exact” for interval estimation of binomial proportions. The American Statistician.

[CR4] Appah, C.K.I. 2003. *Nominal Derivation in Akan*. *A Descriptive Analysis.* M.A. Thesis, Norwegian University of Science and Technology, Norway.

[CR5] Arciuli J (2017). The multi-component nature of statistical learning. Philosophical Transactions of the Royal Society B.

[CR6] Arciuli J, Simpson IC (2012). Statistical learning is related to reading ability in children and adults. Cognitive Science.

[CR7] Aslin RN, Newport EL (2014). Distributional language learning: Mechanisms and models of category formation. Language Learning.

[CR8] Aslin RN, Saffran JR, Newport EL (1998). Computation of conditional probability statistics by 8-month-old infants. Psychological Science.

[CR9] Baguley T (2009). Standardized or simple effect size: What should be reported?. British Journal of Psychology.

[CR10] Baguley T (2012). Serious Stats.

[CR11] Baker CI, Olson CR, Behrmann M (2004). Role of attention and perceptual grouping in visual statistical learning. Psychological Science.

[CR12] Barr DJ, Levy R, Scheepers C, Tily HJ (2013). Random effects structure for confirmatory hypothesis testing: Keep it maximal. Journal of Memory and Language.

[CR13] Bates D, Maechler M, Bolker B, Walker S (2015). Fitting linear mixed- effects models using lme4. Journal of Statistical Software.

[CR14] Batterink LJ, Reber PJ, Neville HJ, Paller KA (2015). Implicit and explicit contributions to statistical learning. Journal of Memory and Language.

[CR15] Bertels J, Boursain E, Destrebecqz A, Gaillard V (2015). Visual statistical learning in children and young adults: How implicit?. Frontiers in Psychology.

[CR16] Berwick RC, Chomsky N (2015). Why Only Us: Language and Evolution.

[CR17] Booij G, van Santen A (2017). Morfologie. De woordstructuur van het Nederlands. 3e geheel herziene druk.

[CR18] Bybee, J. & J.L. McClelland. (2005). Alternatives to the combinatorial paradigm of linguistic theory based on domain general principles of human cognition. In N.A. Ritter (ed.), *The Role of Linguistics in Cognitive Science,* Special Issue of *The Linguistic Review*, 22(2–4), 381–410.

[CR19] Casenhiser D, Goldberg AE (2005). Fast mapping between a phrasal form and meaning. Developmental Science.

[CR20] Chen CH, Gershkoff-Stowe L, Wu CY, Cheung H, Yu C (2017). Tracking multiple statistics: Simultaneous learning of object names and categories in English and Mandarin speakers. Cognitive Science.

[CR21] Chomsky N (1965). Aspects of the Theory of Syntax.

[CR22] Cleeremans A, Banerjee R, Chakrabarti BK (2008). Consciousness: The radical plasticity thesis. Progress in Brain Research.

[CR23] Cleeremans A (2011). The radical plasticity thesis: How the brain learns to be conscious. Frontiers in Psychology.

[CR24] Cleeremans A (2014). Connection conscious and unconscious processing. Cognitive Science.

[CR25] Conway CM, Christiansen MH (2006). Statistical learning within and between modalities: Pitting abstract against stimulus-specific representations. Psychological Science.

[CR26] Conway CM, Bauernschmidt A, Huang SS, Pisoni DB (2010). Implicit statistical learning in language processing: Word predictability is the key. Cognition.

[CR27] Culbertson J, Newport EL (2015). Harmonic biases in child learners: In support of language universals. Cognition.

[CR28] Culbertson J, Schuler K (2019). Artificial language learning in children. Annual Review of Linguistics.

[CR29] Endress AD, Bonatti LL (2007). Rapid learning of syllable classes from a perceptually continuous speech stream. Cognition.

[CR30] Erickson LC, Thiessen ED (2015). Statistical learning of language: Theory, validity and predictions of a statistical learning account of language acquisition. Developmental Review.

[CR31] Evans JL, Saffran JR, Robe-Torres K (2009). Statistical learning in children with specific language impairment. Journal of Speech, Language, and Hearing Research.

[CR32] Frank MC, Goodmann ND, Tenenbaum JB (2009). Using speakers’ referential intentions to model early cross-situational word learning. Psychological Science.

[CR33] Frank MC, Tenenbaum JB, Fernald A (2013). Social and discourse contributions to the determination of reference in cross-situational word learning. Language Learning and Development.

[CR34] Frost RLA, Monoghan P (2016). Simultaneous segmentation and generalization of non-adjacent dependencies from continuous speech. Cognition.

[CR35] Gabay Y, Thiessen ED, Holt LL (2015). Impaired statistical learning in developmental dyslexia. Journal of Speech, Language, and Hearing Research.

[CR36] Gelman A, Hill J (2007). Data Analysis Using Regression and Multilevel/Hierarchical Models.

[CR37] Goldberg AE, Casenhiser D, Sethuraman N (2004). Learning argument structure generalizations. Cognitive Linguistics.

[CR38] Goldberg AE, Casenhiser D, White TR (2007). Constructions as categories of language. New Ideas in Psychology.

[CR39] Gómez RL (2002). Variability and detection of invariant structure. Psychological Science.

[CR40] Gomez RL, Gerken L (1999). Artificial grammar learning by 1-year-olds leads to specific and abstract knowledge. Cognition.

[CR41] Hochman J-R, Endress AD, Mehler J (2010). Word frequency as a cue for identifying function words in infancy. Cognition.

[CR42] Jaeger, T. F. Post to HLP/Jaeger lab blog. May 14 2009, https://hlplab.wordpress.com/2009/05/14/random-effect-structure/

[CR43] Kachergis G, Yu C, Shiffrin RM (2014). Cross-situational word learning is both implicit and strategic. Frontiers in Psychology.

[CR44] Kidd E (2011). Implicit statistical learning is directly associated with the acquisition of syntax. Developmental Psychology.

[CR45] Kruskal W, Mosteller F (1979). Representative sampling: 3: Current statistical literature. International Statistical Review.

[CR46] Kukull W, Ganguli M (2012). Generalizability. The trees, the forest, and the low-hanging fruit. Neurology.

[CR47] Lammertink I, Boersma P, Wijnen F, Rispens J (2017). Statistical learning in specific language impairment: A meta-analysis. Journal of Speech, Language, and Hearing Research.

[CR48] Lany J (2014). Judging words by their covers and the company they keep: Probabilistic cues support word learning. Child Development.

[CR49] Lany J, Saffran JR, Rubenstein JLR, Rakic P (2013). Statistical learning mechanisms in infancy. Comprehensive Developmental Neuroscience: Neurel Circuit Development and Function in the Brain.

[CR50] Lew-Williams C, Pelucchi B, Saffran JR (2011). Isolated words enhance statistical language learning in infancy. Developmental Science.

[CR51] Lidz J, Gagliardi A (2015). How nature meets nurture: Universal grammar and statistical learning. Annual Review of Linguistics.

[CR52] Mintz TH, Newport EL, Bever TG (2002). The distributional structure of grammatical categories in speech to young children. Cognitive Science.

[CR53] Monaghan P, Mattaock K, Davies RAI, Smith AC (2015). Gavagai is as Gavagai does: Learning nouns and verbs from cross-situational statistics. Cognitive Science.

[CR54] Parnes, A.T. Ang marks the what? An analysis of noun phrase markers in Cebuano. M.A. Thesis, Yale University, Connecticut.

[CR55] Perruchet P, Pacton S (2006). Implicit learning and statistical learning: One phenomenon, two approaches. Trends in Cognitive Science.

[CR56] Perruchet P, Vinter A (1998). PARSER: A model for word segmentation. Journal of Memory and Language.

[CR57] Pinker S (1994). The Language Instinct.

[CR58] Pinker S (1997). How the Mind Works.

[CR59] Pinto M, Zuckerman S (2018). Coloring book: A new method for testing language comprehension. Behavior Research Methods.

[CR60] Powell, M.J.D., (2009) The BOBYQA algorithm for bound constrained optimization without derivatives. Technical Report, Department of Applied Mathematics and Theoretical Physics, University of Cambridge.

[CR61] Psychology Software Tools, Inc. (2012). E-Prime 3.0. Retrieved from http://www.pstnet.com/.

[CR62] Qian, T., Reeder, P.A., Aslin, R.N., Tenenbaum, J.B., & Newport, E.L., Exploring the role of representation in models of grammatical category acquisition. In Proceedings of the annual meeting of the cognitive science society, vol. 34, pp. 881–886.

[CR63] R Core Team. 2015. R: A language and environment for statistical computing. R Foundation for Statistical Computing, Vienna, Austria. Retrieved from https://www.R-project.org/.

[CR64] Raviv L, Arnon I (2018). Systematicity, but not compositionality: Examining the emergence of linguistic structure in children and adults using iterated learning. Cognition.

[CR65] Reber A (1967). Implicit learning of artificial grammars. Journal of Verbal Learning and Verbal Behavior.

[CR66] Rebuschat P (2013). Measuring implicit and explicit knowledge in second language research. Language Learning.

[CR67] Rebuschat P, Williams JN, Rebuschat P, Williams JN (2012). Introduction: Statistical learning and language acquisition. Statistical Learning and Language Acquisition.

[CR68] Romberg AR, Saffran JR (2010). Statistical learning and language acquisition. Wiley Interdisciplinary Reviews: Cognitive Science.

[CR69] Saffran JR, Johnson EK, Aslin RN (1996). Word-segmentation: The role of distributional cues. Journal of Memory and Language.

[CR70] Samara A, Smith K, Brown H, Wonnacott E (2017). Acquiring variation in an artificial language: Children and adults are sensitive to socially-conditioned linguistic variation. Cognitive Psychology.

[CR71] Smith LB, Yu C (2008). Infants rapidly learn word-referent mappings via cross-situational statistics. Cognition.

[CR72] Smith LB, Suanda SH, Yu C (2014). The unrealized promise of infant statistical word-referent learning. Trends in Cognitive Science.

[CR73] Sociaal Cultureel Planbureau [SCP]. (2016). Sociaal-economische status per postcodegebied.

[CR74] Spit S, Andringa S, Rispens J, Aboh EO (2019). The opt out paradigm. First steps towards a new experimental method that measures meta-linguistic awareness. Dutch Journal of Applied Linguistics.

[CR75] Spit, S., Andringa, S., Rispens, J., Aboh, E.O., & Vet, D.J., (2019b). Meta-linguistic awareness in early (second) language acquisition*.* Retrieved from osf.io/bp5qe. 10.17605/OSF.IO/BP5QE

[CR76] Swingley D (2005). Statistical clustering and the contents of the infant vocabulary. Cognitive Psychology.

[CR77] Tal, S., & Arnon, I., (2020). Do children preferentially mark unpredictable material? The case of optional plural marking. In Proceedings of the 42^nd^ Annual Conference of the Cognitive Science Society, pp. 3226–3232.

[CR78] Thiessen ED, Kronstein AT, Hufnagle DG (2013). The extraction and integration framework: A two-process account of statistical learning. Psychological Bulletin.

[CR79] Timmermans B, Cleeremans A, Overgaard M (2015). How can we measure awareness? An overview of current methods. Behavioural Methods in Consciousness Research.

[CR80] Tomasello M (2003). Constructing A Language: A Usage-Based Theory of Language Acquisition.

[CR81] Toro JM, Sinnett S, Soto-Faraco S (2005). Speech segmentation by statistical learning depends on attention. Cognition.

[CR82] Turk-Browne NB, Jungé J, Scholl BJ (2005). The automaticity of visual statistical learning. Journal of Experimental Psychology.

[CR83] Ullman M, Hickok G, Small SA (2016). The declarative/procedural model: A neurobiological model of language learning, knowledge and use. The Neurobiology of Language.

[CR84] van Witteloostuijn M, Boersma P, Wijnen F, Rispens J (2017). Visual artificial grammar learning in dyslexia: A meta-analysis. Research in Developmental Disabilities.

[CR85] Vlach HA, Johnson SP (2013). Memory constraints on infants’ cross-situational statistical learning. Cognition.

[CR86] Vouloumanos A (2008). Fine-grained sensitivity to statistical information in adult word learning. Cognition.

[CR87] Wonnacott E, Boyd JK, Thomson J, Goldberg AE (2012). Input effects on the acquisition of a novel phrasal construction in 5 year olds. Journal of Memory and Langauge.

[CR88] Wonnacott E, Brown H, Nation K (2017). Skewing the evidence: The effect of input structure on child and adult learning of lexically based patterns in an artificial language. Journal of Memory and Language.

[CR89] Yang C, Montrul S (2017). Learning datives: The tolerance principle in monolingual and bilingual acquisition. Second Language Research.

[CR90] Yu C, Smith LB (2007). Rapid word learning under uncertainty via cross-situational statistics. Psychological Science.

[CR91] Yurovsky D, Smith LB, Yu C (2013). Statistical word learning at scale: The baby's view is better. Developmental Science.

